# The Chemically Synthesized Ageladine A-Derivative LysoGlow84 Stains Lysosomes in Viable Mammalian Brain Cells and Specific Structures in the Marine Flatworm *Macrostomum lignano*

**DOI:** 10.3390/md13020920

**Published:** 2015-02-11

**Authors:** Thorsten Mordhorst, Sushil Awal, Sebastian Jordan, Charlotte Petters, Linda Sartoris, Ralf Dringen, Ulf Bickmeyer

**Affiliations:** 1 Alfred-Wegener-Institut, Helmholtz-Zentrum für Polar- und Meeresforschung, Am Handelshafen 12, Bremerhaven 27570, Germany; E-Mails: Thorsten.Mordhorst@awi.de (T.M.); Jordan.Sebastian@gmx.de (S.J.); linda_sartoris@web.de (L.S.); 2 Marnas Biochemicals GmbH, Parkstraße 5, Bremerhaven 27580, Germany; 3 Center for Biomolecular Interactions, Faculty 2 (Biology/Chemistry), University of Bremen, PO. Box 330440, Bremen 28334, Germany; E-Mails: sush01wal@gmail.com (S.A.); petters@uni-bremen.de (C.P.); ralf.dringen@uni-bremen.de (R.D.); 4 Hochschule Bremerhaven, An der Karlstadt 8, Bremerhaven 27568, Germany; 5 Freie Universität Berlin, Institut für Biologie, Schwendenerstr. 1, Berlin 14195, Germany

**Keywords:** ageladine A derivative, fluorescence, live imaging, lysosomes, new dye

## Abstract

Based on the chemical structure and the known chemical synthesis of the marine sponge alkaloid ageladine A, we synthesized the ageladine A-derivative 4-(naphthalene-2-yl)-*1H*-imidazo[4,5-*c*]pyridine trifluoroacetate (LysoGlow84). The two-step synthesis started with the Pictet-Spengler reaction of histamine and naphthalene-2-carbaldehyde to a tetrahydropyridine intermediate, which was dehydrogenated with activated manganese (IV) oxide to LysoGlow84. Structure and purity of the synthesized LysoGlow84 were confirmed by NMR spectroscopy and mass spectrometry. The fluorescence intensity emitted by LysoGlow84 depended strongly on the pH of the solvent with highest fluorescence intensity recorded at pH 4. The fluorescence maximum (at 315 nm excitation) was observed at 440 nm. Biocompatibility of LysoGlow84 was investigated using cultured rat brain astrocytes and the marine flatworm *Macrostomum lignano*. Exposure of the astrocytes for up to 6 h to micromolar concentrations of LysoGlow84 did not compromise cell viability, as demonstrated by several viability assays, but revealed a promising property of this compound for staining of cellular vesicles. Conventional fluorescence microscopy as well as confocal scanning microscopy of LysoGlow84-treated astrocytes revealed co-localization of LysoGlow84 fluorescence with that of LysoTracker^®^ Red DND-99. LysoGlow84 stained unclear structures in *Macrostomum lignano*, which were identified as lysosomes by co-staining with LysoTracker. Strong fluorescence staining by LysoGlow84 was further observed around the worms’ anterior gut and the female genital pore which were not counterstained by LysoTracker Red. Thus, LysoGlow84 is a new promising dye that stains lysosomes and other acidic compartments in cultured cells and in worms.

## 1. Introduction

Fluorescent small molecules are present in the bright, dark and twilight zones of the oceans, produced by and transported in many different organisms. They fulfill manifold ecological and behaviorally relevant purposes in marine organisms [1]. Aquatic species mostly emit fluorescence in the blue/green wavelength range, as 470 nm light travels the longest distance and blue/UV light reaches deep into open waters, due to the physical properties of light scattering and transmission in water [2]. This is why many marine fluorescent compounds have similar optical properties with conspicuous blue light fluorescence.

**Figure 1:**
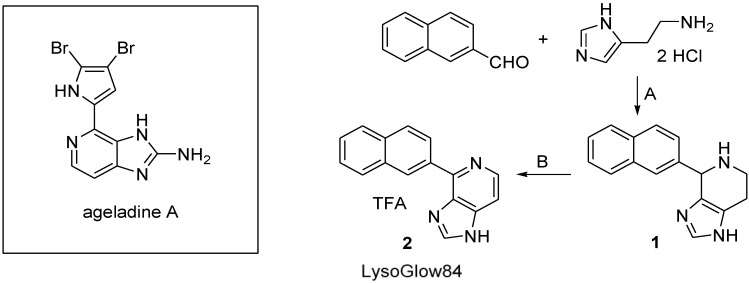
Structure of ageladine A and synthesis of 4-(naphthalene-2-yl)-*1H*-imidazo[4,5-*c*]pyridine trifluoroacetate (structure 2, LysoGlow84): **A**: KOH, EtOH, 24 h, 80 °C, 99%; **B**: (1) MnO_2_, pyridine, acetone, 18 h, 80 °C; (2) TFA, EtOH, diethyl ether, 71%.

The fluorescing alkaloid ageladine A (Figure 1) was initially isolated from the sponge Agelas in search for angiogenesis inhibitors. The chemical structure of ageladine A was identified as brominated pyrrole-imidazole alkaloid [3]. Ageladine A shows a pH-dependent fluorescence with an emission maximum in the blue wavelength range [4,5] and has successfully been used to stain acidic vesicles in mouse hippocampal neurons [6]. The successful chemical synthesis of ageladine A was reported in 2006 by Karuso and Weinreb [7,8]. We have based our synthesis on the one published by Karuso [7] and modified by Ando [9], to generate a set of structurally related derivatives of ageladine A and to investigate such derivatives as potentially useful tools for fluorescence staining of cellular compartments with different pH milieus.

Lysosomes are small cellular vesicles which contain hydrolyzing enzymes such as lipases, proteases and nucleases. These organelles are characterized by a low pH (pH = 4.5–5) which gives the digesting enzymes an optimal catalytic environment. Microorganisms, macromolecules, and organelles are taken up or fused with lysosomes in order to hydrolyze them. Lysosomes can therefore be considered as “recycling units of a cell” [10]. Lysosomes are present in all eukaryotic cells and play a major role in their physiology. As endosomes, synaptic vesicles and transport vesicles are acidified during the process of vesicle recycling, new dyes for a better detection and live imaging of acidic vesicles are highly warranted. Especially new dyes with acidic pH optima and new spectral characteristics have the potential to serve as new tools in vesicle related research.

The fluorescence of the new compound LysoGlow84 (Figure 1) was compared to the commercially available dye LysoTracker^®^ Red DND-99 (Invitrogen, Darmstadt, Germany) in isolated mammalian astrocytes as well as in the living and intact flatworms *Macrostomum lignano*. This marine transparent flatworm was first described in 2005 [11] and can be considered as a model species for physiological investigations of marine invertebrates. *Macrostomum lignano* has previously been used in live imaging studies to answer questions regarding physiological response to environmental stress [12]. The worm’s transparent habitus allows to address physiological questions in intact animals, which is physiologically preferable over use of dissected or sliced tissues [4,13].

Here we report that the ageladine A derivative LysoGlow84 possesses a strong pH-dependent fluorescence and causes no acute cell toxicity. LysoGlow84 staining of lysosomes in cultured cells and acidic compartments in the flatworm *Macrostomum lignano* suggests that this dye is suitable for live imaging of acidic compartments* in vitro* and* in vivo*. 

## 2. Results and Discussion

### 2.1. Synthesis of LysoGlow84

In a two-step reaction 4-(naphthalene-2-yl)-*1H*-imidazo[4,5-*c*]pyridine trifluoroacetate (Figure 1 structure 2; LysoGlow84) was synthesized, as shown in Figure 1, following the strategy previously used to synthesize compounds that are structurally related to the marine natural product ageladine A [14].

Starting with the Pictet-Spengler reaction of histamine dihydrochloride and naphthalene-2-carbaldehyde, using potassium hydroxide as base in refluxing ethanol, [9,15,16] the resulting 4-(naphthalene-2-yl)-4,5,6,7-tetrahydro-*1H*-imidazo[4,5-*c*]pyridine **1** was obtained as a mixture of enantiomers in nearly quantitative yield after chromatographic purification. To keep the subsequent oxidation step as simple as possible, a dehydrogenation step with activated manganese (IV) oxide was chosen [17,18], which allowed easy separation of the dehydrogenating agent by filtration of the reaction mixture. Other prominent agents that are commonly used to dehydrogenate tetrahydropyridine rings to aromatic systems, such as chloranil, Pd/C or IBX [19], had disadvantages during the separation process and were therefore not considered for further optimization of the synthesis method. Manganese (IV) oxide dehydrogenates the intermediate **1** in the presence of pyridine. Acetone as a non-oxidizable solvent with an easy to handle boiling point turned out to be the best choice of solvent for the reaction. Although the educt **1** is hardly soluble in acetone, the product forming during the reaction process is easily dissolved. The final product of the synthesis, 4-(naphthalene-2-yl)-*1H*-imidazo[4,5-*c*]pyridine (**2a**) was obtained in 71% yield after chromatographic purification. The salt of the oxidization product was found to be more stable and easier to handle than the free base. The salt was quantitatively precipitated using ethanolic hydrochloric acid or trifluoroacetic acid before decreasing the polarity of the solvent with a large volume of diethyl ether.

The final product 4-(naphthalene-2-yl)-*1H*-imidazo[4,5-*c*]pyridine trifluoroacetate (compound **2**, Figure 1) is a stable, non-hygroscopic dye that shows no sign of decomposition at or below room temperature during long term storage over several months. The molecular mass and the chemical structure of LysoGlow84 were confirmed by mass spectrometry and NMR analysis.

**Figure 2:**
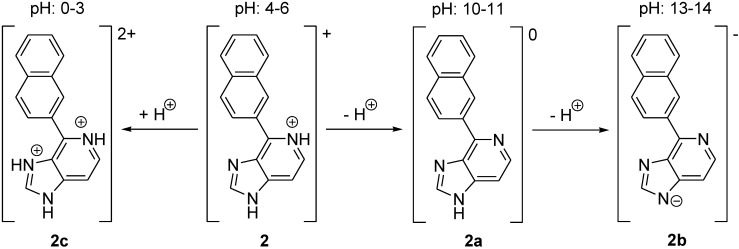
Deprotonation and protonation of 4-(naphthalene-2-yl)-1H-imidazo[4,5-*c*]pyridine (LysoGlow84).

LysoGlow84 dissolves readily in water, methanol or ethanol. The solubility in water is low at a pH of around 10 (pH 9–12) as these conditions cause the deprotonation of compound **2** to the less polar compound **2a**. Above pH 12 the deprotonation of the last nitrogen-bound hydrogen is favored, which generates the negatively charged compound **2b** that dissolves easily (Figure 2). At pH values lower than 3 (**2c**) the molecule has two positive charges, dissolves in water but shows only weak fluorescence.

### 2.2. Stability of LysoGlow84 in Solution

In order to investigate the stability of LysoGlow84 in solution, a long-term experiment (5 weeks) was carried out. LysoGlow84 was dissolved in deuterium oxide and deuterated methanol at a concentration of 10 g/L, respectively. Twice a week the NMR spectrum of each solution was measured. During this period the NMR-tubes were stored at room temperature and exposed to daylight. No decomposition of the molecule occurred in either of the two solvents.

### 2.3. Fluorescence Spectra Vary with pH Values

**Figure 3:**
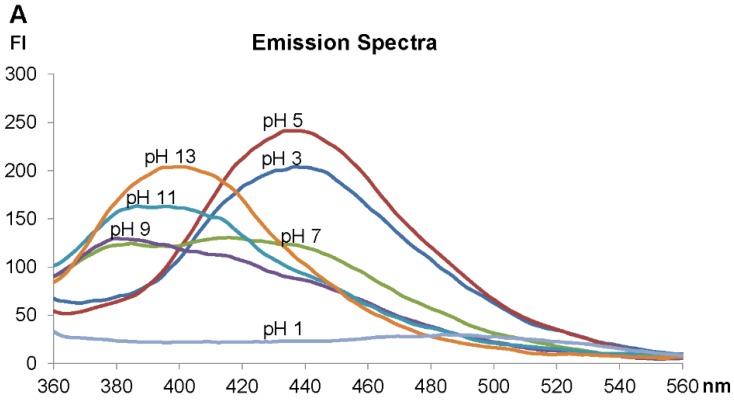

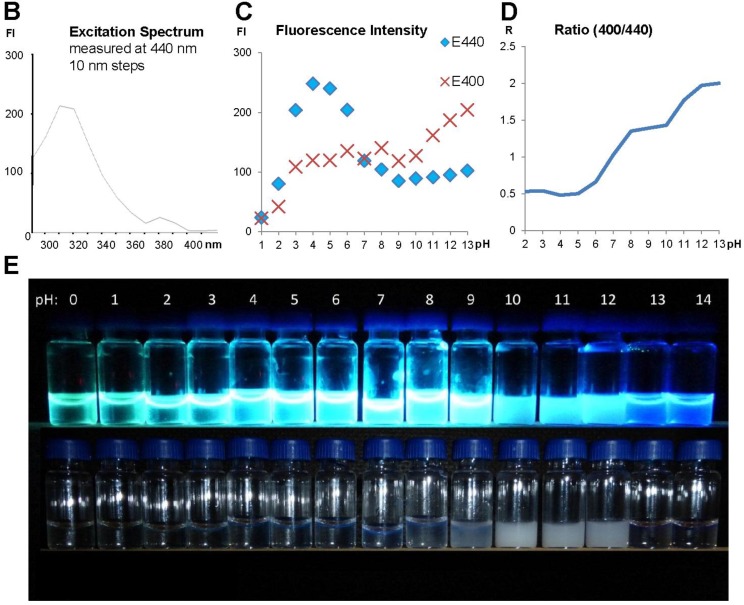
Fluorescence properties of LysoGlow84. **A**: Emission wavelengths of LysoGlow84 in dependency of the pH value in solutions containing 9 g/L NaCl. Intensity is given in arbitrary fluorescence units (FI) (exc. wavelength 320 nm). **B**: Excitation spectrum. **C**: Fluorescence measured at 400 nm and 440 nm with different pH values. **D**: Ratio of intensities at emission wavelength 400 nm and 440 nm in dependency of the pH. **E**: LysoGlow84 in water with different pH values during UV excitation (above) and with daylight (below) demonstrates its pH-dependent fluorescence and turbidity.

**Figure 4:**
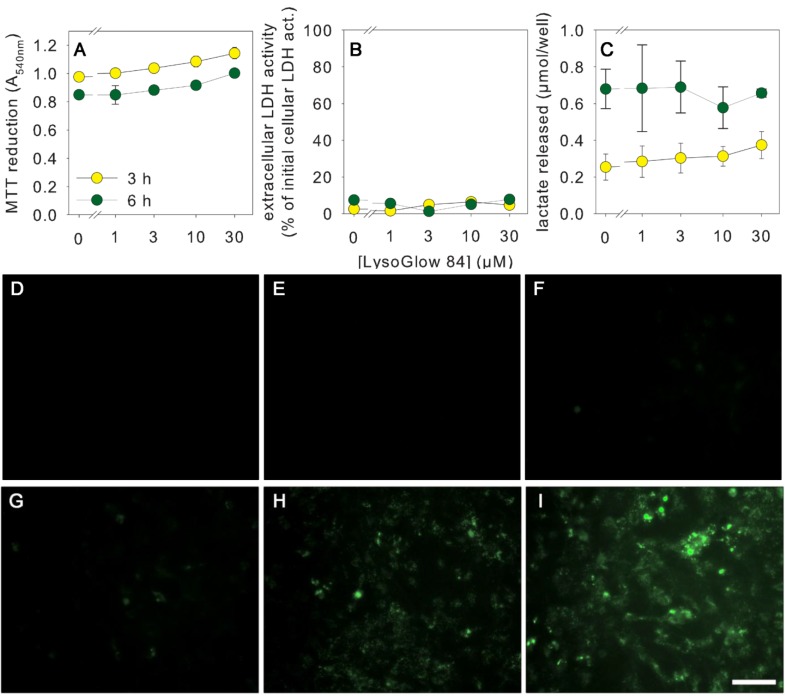
Test for acute toxicity and cellular staining of cultured astrocytes with LysoGlow84. The cells were incubated for 3 h (**A**–**I**) or 6 h (**A**–**C**) with the indicated concentrations of LysoGlow84 or with the vehicle control (DMSO). Cellular MTT reduction capacity (**A**), extracellular lactate dehydrogenase (LDH) activity (**B**) and extracellular lactate content (**C**) were determined. ANOVA revealed no significant difference between treatment groups. Incubation with DMSO in an amount representing the DMSO content applied with the highest LysoGlow84 concentration was for none of the parameters significantly altered (data not shown). Panel **D**–**I** show the fluorescence staining of the cultures after incubation without LysoGlow84 (controls without (**D**) or with DMSO (**E**)) or with LysoGlow84 in concentrations of 1 (**F**), 3 (**G**), 10 (**H**) or 30 µM (**I**). The scale bar in panel I represents 50 µm and applies to the panels **D**–**I**.

Analysis of the fluorescence spectra of the ageladine A derivative LysoGlow84 revealed maximal excitation at 315 nm (Figure 3B). In acidified water an emission maximum of LysoGlow84 was observed at 440 nm, whereas in alkaline water the emission maximum shifted to shorter wavelengths (380–400 nm) (Figure 3A). The recorded excitation spectra are broad and cover a wide nm range, with strong excitation at 290–350 nm, which diminishes at 400 nm (Figure 3B). Emission is strong between 360 nm and 520 nm with a peak at 440 nm (Figure 4A). The intensity of fluorescence depends strongly on the pH of the solvent with maximal values determined between pH 4 and 5. The emission maximum was shifted from 440 nm at pH 2–6 to 400 nm at pH 10–13. LysoGlow84 is poorly soluble in water at pH values between 8 and 12 (Figure 3E), most likely because it is present as uncharged compound in this pH range (Figure 2). At pH 13 LysoGlow84 completely dissolves again, most likely by additional deprotonation to a negatively charged soluble molecule (Figure 2). At pH values lower than pH 3, the molecule is double charged (Figure 2) but only weakly fluorescent (Figure 3). It is important to note that there are two isosbestic points at ~405 nm for pH 3–9 and another at ~450 nm for pH 9–13, marking two pH insensitive wavelengths. In Figure 3D two pKa values at pH 7 and pH 11.5 become apparent, indicating three states of the molecule between pH 3 and pH 13. At low pH (<3) the fluorescence intensity decreases dramatically (see Figure 3C) which is likely caused by a change in the molecular structure through further protonation (Figure 2, structure **2c**). Cleavage or other destruction of the molecule is unlikely to be initiated by a higher proton concentration or at high pH. We therefore propose four possible protonation states of LysoGLow84 which depend on the pH (Figure 2).

Ionic strength and ion composition have only marginal effects on the fluorescence excitation and emission spectra of LysoGlow84 (data not shown).

A shift in the emission maximum can be used to calculate a relationship between pH and R = I400/I440. I400 and I440 give fluorescence intensities at 400 nm and 440 nm, respectively. The ratio R strongly depends on the pH range and reaches from R = 0.5 at pH 2–pH 5 to R = 2 at pH 12. The highest resolution of R is between pH 6 and pH 8 and between pH 10 and pH 12. There is no linear relationship between pH and R value, but R can be used to determine the pH of a medium over a wide pH range with varying accuracy (Figure 3D).

### 2.4. Effects of LysoGlow84 on Cultured Primary Astrocytes

To test for potential toxicity of LysoGlow84 towards mammalian cells, primary rat astrocyte cultures were incubated for 3 h or 6 h at 37 °C with or without LysoGlow84 in concentrations between 1 and 30 µM in the incubation buffer (IB). As controls, the cells were incubated without LysoGlow84 but with DMSO at a final concentration equaling the DMSO content of the media of cells exposed to 30 µM LysoGlow84. None of the applied control and treatment conditions altered the morphology of the cells (data not shown) or compromised cell viability, as demonstrated by the absence of a significant decline in MTT reduction capacity (Figure 4A), by the absence of a significant increase in extracellular LDH activity (Figure 4B), and by the absence of an alteration in cellular lactate generation and release (Figure 4C). These results demonstrate that LysoGlow84 applied at concentrations of up to 30 µM does not acutely compromise the viability of cultured primary astrocytes. The absence of an increase in extracellular LDH activity demonstrates maintenance of membrane integrity during treatment, whereas the absence of alterations in MTT reduction capacity and glycolytic lactate production demonstrate that the basal metabolism of the cells was not affected by treatment with LysoGlow84. 

To investigate whether cultured astrocytes can be stained by LysoGlow84, the cells were incubated for 3 h at 37 °C with LysoGlow84 in concentrations ranging from 1 µM to 30 µM and the cellular fluorescence emitted at 420 nm was documented using a fluorescence microscope with UV light excitation at 330–380 nm. Cells incubated without LysoGlow84 did not show substantial auto-fluorescence under the applied conditions (Figure 4D). In contrast, astrocytes that had been incubated with LysoGlow84 showed a concentration-dependent increase in cells fluorescence that was weak for cells treated with 1 µM (Figure 4F) or 3 µM LysoGlow84 (Figure 4G) but strong for cells exposed to 10 µM (Figure 4H) or 30 µM LysoGlow84 (Figure 4I).

**Figure 5:**
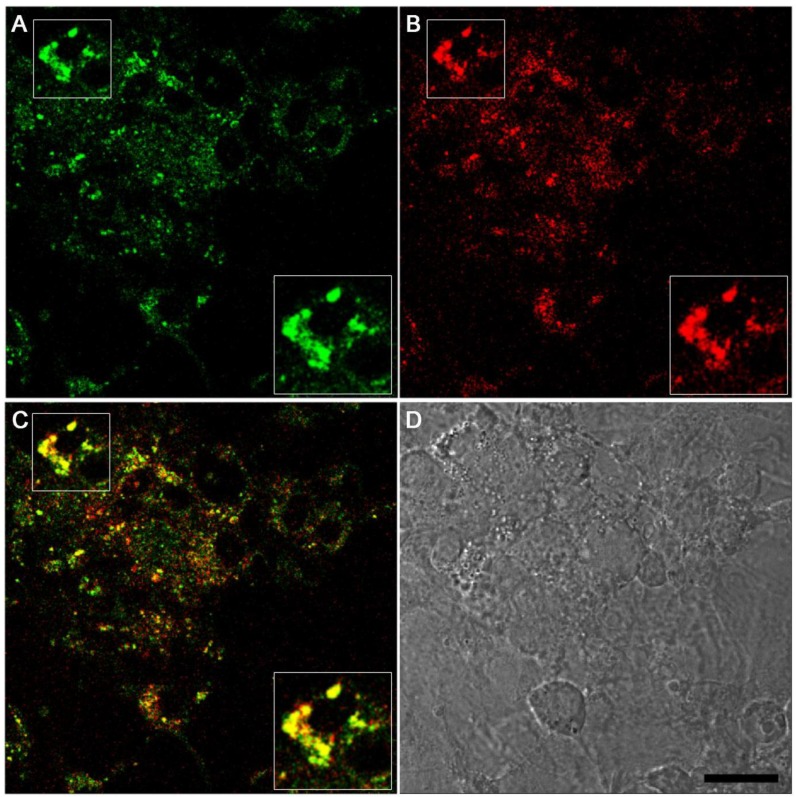
Confocal images of LysoGlow84 and LysoTracker Red fluorescence in cultured astrocytes. The cells were incubated with 10 µM LysoGlow84 and 70 nM of LysoTracker Red for 3 h at 37 °C. Images of LysoGlow84 fluorescence (**A**), LysoTracker Red fluorescence (**B**), and the overlay (**C**) which shows co-localization of both fluorescent dyes in yellow (highlighted in insets). Panel **D** shows the transmission light image of the cells. The scale bar in **D** represents 20.1 µm and applies to all images.

High magnification of LysoGlow84-stained astrocytes revealed that the observed fluorescence had a spotted pattern, suggesting vesicular localization of the fluorescent dye in the cells (Figure 5A). Due to this spotted staining pattern (Figure 5) and the high fluorescence of the compound at pH 4–6 (Figure 3), it was hypothesized that the accumulated LysoGlow84 might be located in lysosomes. Indeed, confocal analysis of astrocytes that had been co-incubated with LysoGlow84 and LysoTracker Red for 3 h at 37 °C revealed a high degree of co-localization of both fluorescent dyes (Figure 5). As LysoGlow84 does not acutely affect the metabolism and the viability of the astrocytes, the cellular fluorescence observed for LysoGlow84-treated astrocytes can be attributed to LysoGlow84 that has been accumulated by viable astrocytes.

### 2.5. Staining of Living Individuals of the Flatworm Macrostomum Lignano with LysoGlow84 and LysoTracker^®^ Red DND-99

**Figure 6:**
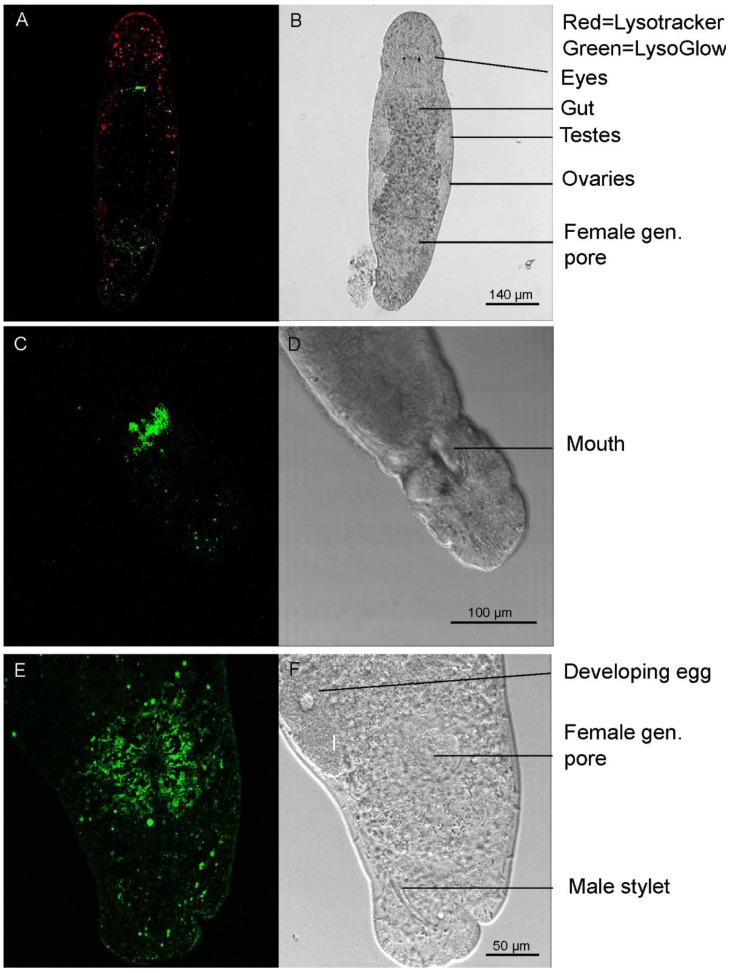
Flatworms of the species *Macrostomum lignano* were exposed for 3 h to 20 µM LysoGlow84 and 60 nM LysoTracker Red. **A**: Fluorescence images of the worm stained with LysoGlow84 (green) and LysoTracker Red (red). **B**: Transmission image for panel A shows well defined organs. **C**: Confocal image (projection) of one individual of *Macrostomum lignano* stained with LysoGlow84. One specific area close to the mouth in the anterior part of the gut is stained. **D**: Transmission image of the worm shown in panel C. **E**: Tissue around the female genital pore, assumed to be glands, stained with LysoGlow84. **F**: Transmission image for panel E showing well defined organs, including the female genital pore, the male stylus, and a developing egg. Scale bars are indicated in the transmission images (**B**, **D**, **F**).

**Figure 7:**
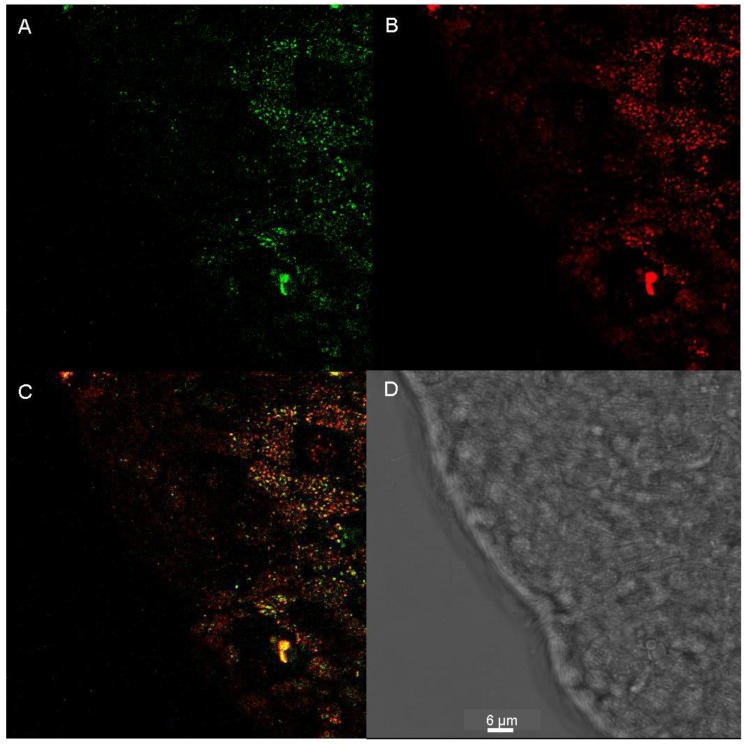
**A**: LysoGlow84 staining of epidermal cell layer of living and intact individual of *Macrostomum lignano* (green). **B**: LysoTracker Red staining (red). **C**: Overlay of the images shown in panels **A** and **B**. **D**: Transmission image of shown images. The scale bar in **D** represents 6 µm and applies to all images.

Living individuals of *Macrostomum lignano* were exposed to LysoGlow84 and LysoTracker Red for 1 h. The worms contained cells that emitted fluorescence derived from both dyes (Figure 6A, Figure 7). Structures with bright fluorescence of LysoTracker Red and LysoGlow84 are most probably rhabdites that have previously been identified as acidic and stained with a pH sensitive dye [5]. However, LysoGlow84 stained more structures in the worm than LysoTracker Red (Figure 6A). Especially regions at the upper end of the gut close to the mouth and around the genital pore were strongly stained by LysoGlow84, but not by LysoTracker Red (Figure 6C,E). Co-staining and simultaneous illumination at both excitation wavelength (340 nm and 561 nm) showed that LysoGlow84 emits minor fluorescence in the red (LysoTracker Red) emission wavelength. However, separate excitation images with only one excitation wavelength for each dye demonstrated a clear separation of staining by the different dyes in both regions of interest. 

Images with higher magnification showed great similarities between the staining of the worm’s epidermal cells with LysoGlow84 and LysoTracker Red (Figure 7A,B)*.* However, the overlay image of both staining patterns revealed incomplete matching of both patterns, although vesicles stained by LysoGlow84 were in most cases also positive for LysoTracker Red (Figure 7C).

Our live imaging experiments with *Macrostomum lignano* showed that some structures in the worm are stained by LysoGlow84, indicating that this compound is taken up by the worms during exposure to the dye in seawater at pH 8.1. Most likely the uncharged lipophilic form **(**Figure 2, structure 2a) of LysoGlow84 (Figure 1), which would be present at normal sea water pH, will cross the cell membranes. In acidic compartments of the cells, LysoGlow84 will be partially protonated and trapped, as is the case with LysoTracker Red [20,21]. This is the likely reason for the co-localization of both dyes in many structures and compartments in the astrocytes and the worms. However, in both, cultured cells and worms, the fluorescence determined for LysoGlow84 and LysoTracker Red did not perfectly match, suggesting that both dyes differ in their potential to stain structures. Wiegand* et al.* [22] stated that LysoTracker Red stains all acidic structures in the cells, which is only partly corroborated by our study assuming that LysoGlow84 stains additional acidic compartments. Differences in cellular uptake, metabolism, pH sensitivity of fluorescence and/or export of LysoGlow84 and LysoTracker Red might contribute to the observed differences in the cellular localization. It also has to be considered that LysoGlow84, a compound derived from a bioactive natural product, could specifically stain some biomolecules and that part of the observed fluorescence signal is independent of the cellular pH.

Attempts to calculate the exact pH values based on LysoGlow84 images of living cells by ratiometric measurements failed so far, because fluorescence emission at 400 nm was insufficient and just above baseline values.

## 3. Experimental Section

### 3.1. Chemicals

Dulbecco’s modified Eagle’s medium (DMEM), penicillin G/streptomycin sulfate solution and LysoTracker^®^ Red DND-99 were purchased from Gibco/Invitrogen (Darmstadt, Germany) and fetal calf serum and penicillin/streptomycin solution from Biochrom (Berlin, Germany). The enzymes glutamate pyruvate transaminase and lactate dehydrogenase (LDH) were purchased from Roche (Mannheim, Germany). NAD^+^ and NADH were from Applichem (Darmstadt, Germany). Deuterated solvents were from Roth (Karlsruhe, Germany). All other chemicals were purchased in analytical grade from Merck (Darmstadt, Germany), Fluka (Buchs, Switzerland), Roth (Karlsruhe, Germany), VWR (Darmstadt, Germany) or Sigma (Steinheim, Germany). 96-well microtiter plates and 24-well plates were from Sarstedt (Nürnberg, Germany). F/2 Medium with silicatef/2-Si enriched [23].

### 3.2. Synthesis and Chemical Characterization of LysoGlow84

Synthesis of 4-(naphthalene-2-yl)-4,5,6,7-tetrahydro-*1H*-imidazo[4,5-*c*]pyridine **1**:

Naphthalene-2-carbaldehyde (0.78 g, 5 mmol), histamine dihydrochloride (0.94 g, 5 mmol) and potassium hydroxide (0.93 g, 16.5 mmol, 3.3 eq.) were dissolved in 120 mL of dry ethanol in a 250 mL round bottom flask. This solution was heated at 80 °C for 24 h. After cooling to room temperature, the solvent was distilled off under reduced pressure, and the remaining residue was chromatographed over silica gel by flash-chromatography, using a gradient of 8:1–1:1 (chloroform/methanol) to yield the product in 99% (1.25 g) as a yellow glass-like solid. ^1^H-NMR (DMSO-d_6_/CDCl_3_ (1:1)) δ 7.65–7.88 (m, 4H), 7.39 (s, 1H), 7.30–7.53 (m, 3H) 5.22 (s, 1H), 2.98–3.20 (m, 2H), 2.54–2.82 (m, 2H) ppm; ^13^C-NMR (DMSO-d_6_/CDCl_3_ (1:1)) δ 139.1, 134.4, 133.1, 133.0, 128.1, 127.9, 127.7, 127.6, 127.2, 126.3, 126.2, 57.4, 41.5, 23.1 ppm. No signals were detected for C-4 and C-5 of the imidazole ring system. *m*/*z* = 499.2606 [2M + H]+ (calc. for C_32_H_31_N_6_^+^: *m*/*z* = 499.2605).

Synthesis of 4-(naphthalene-2-yl)-1H-imidazo[4,5-*c*]pyridine trifluoroacetate (LysoGlow84):

In a 250 mL round bottom flask, 4-(naphthalene-2-yl)-4,5,6,7-tetrahydro-*1H*-imidazo[4,5-*c*]pyridine **1**(1.18 g, 4.7 mmol), activated manganese(IV) oxide (11.75 g, 135 mmol, 25 eq.) and 2.2 mL pyridine (28 mmol, 6 eq.) were dissolved in 100 mL of acetone. This solution was heated at 80 °C for 18 h. After cooling to room temperature, the black mixture was filtered through a celite pad and a bright yellow filtrate was received. The filter cake was additionally washed with acetone/methanol mixture (1:1) until the filtrate was colorless. The solvents were distilled off under reduced pressure, and the residue was chromatographed over silica gel by flash chromatography, using a gradient of 8:1–1:1 (chloroform/methanol) to yield the product as pale orange solid showing bright blue fluorescence at 366 nm. The product **2a** was dissolved in 5 mL of tetrahydrofurane without intermediate characterization. Trifluoroacetic acid (1 mL) was added and the final product was precipitated by addition of diethyl ether until no further solid formed. The trifluoroacetate was yielded in 71% as yellow solid. Analysis of the product by NMR and mass spectrometry revealed the following parameters: ^1^H-NMR (DMSO-d_6_) δ 9.04 (s, 1H), 8.82 (s, 1H), 8.55 (d, 1H), 8.48 (d, 1H), 8.16 (s, 1H), 8.09 (d, 1H), 8.03 (d,1H), 7.97 (m, 2H) ppm; ^13^C-NMR (DMSO-d_6_) δ 149.1, 144.7, 144.1, 137.6, 135.6, 134.4, 132.8, 131.2, 129.5, 129.0, 128.8, 128.6, 128.3, 127.7, 126.4, 109.9 ppm. *m*/*z* = 491.1984 [2M + H]^+^ (calc. for C_32_H_23_N_6_^+^: *m*/*z* = 419.1978).

All NMR spectra were recorded using a Bruker Avance 400 MHz spectrometer at 400 MHz (^1^H NMR) and 100 MHz (^13^C NMR). All experiments were carried out at 300 K using standard parameters. The chemical shifts of the ^1^H- and ^13^C-NMR spectra are reported in ppm relative to internal tetramethylsilane (δ = 0.00 ppm). High resolution mass spectra were recorded with a direct injection ESI-TOF mass spectrometer (Bruker micrOTOF, Bremen, Germany).

The presence of one trifluoroacetate counter ion within salt **2** was confirmed by a NMR titration of **2** in deuterated methanol against sodium methanolate. Two deprotonation steps were observed as shown in Figure 2, starting with the positively charged **2**, passing the neutral molecule **2a** and resulting in the negatively charged **2b**.

### 3.3. Characterization of LysoGlow84 as Fluorescent Molecule

Fluorescence spectra were measured using a Luminescence Spectrometer LS50b (Perkin Elmer). 10^−8^ M LysoGlow84 was dissolved in following solutions. Solvents were: NaCl (30 g/L); LNaCl (10 g/L); artificial sea water NaCl (7.3 g/L); KCl (0.186 g/L); MgCl_2_·6H_2_O (0.203 g/L); CaCl_2_·2H_2_O (0.294 g/L); and NaHPO_4_·H_2_O (0.179 g/L); and MilliQ water. Solutions were adjusted with sodium hydroxide or hydrochloric acid to establish specific pH values.

Fluorescence of LysoGlow84 dissolved in water at different pH values (Figure 2E) was visualized in a small dark chamber (Figure 2E) equipped with a Xenon lamp for UV excitation (maximal emission 366 nm). 

### 3.4. Cell Culture Experiments on Cultured Astrocytes

Primary astrocyte cultures were prepared from the brains of newborn Wistar rats as previously described [24,25]. The harvested brain cells were seeded in culture medium (90% DMEM, 10% fetal calf serum, 18 units/mL penicillin G, 18 μg/mL streptomycin sulfate, 1 mM sodium pyruvate) at a density of 300,000 viable cells in 1 mL medium per well of 24-well plates and were cultured with 10% CO_2_ in the humidified atmosphere in a Sanyo incubator (Osaka, Japan). The culture medium was renewed every seventh day. For experiments, confluent cultures of an age between 13 to 17 days were used.

If not stated otherwise, the cultures were washed with 200 µL of pre-warmed (37 °C) incubation buffer (IB; 145 mM NaCl, 30.4 mM KCl, 1.8 mM CaCl_2_, 1 mM MgCl_2_, 0.8 mM Na_2_HPO_4_, 20 mM HEPES, 5 mM glucose, pH = 7.4) and incubated for 3 h with 200 µL IB containing LysoGlow84 and/or other compounds as indicated. Incubations were terminated by washing the cells twice with 1 mL ice-cold phosphate buffered saline (PBS; 10 mM potassium phosphate buffer, pH = 7.4, containing 150 mM NaCl).

Following treatment, viability of astrocytes was assessed by determining the extracellular activity of the cytosolic enzyme lactate dehydrogenase (LDH), the cellular MTT reduction capacity, and the release of glycolytically generated lactate. The extracellular LDH activity was measured by a microtiter plate-based photometric assay as previously reported [25] and is given as percent of the initial cellular LDH activity (determined in untreated cells that had been lysed with 1% (w/v) Triton X-100 in IB). MTT reduction capacity was determined by a modification of a previously described method [26]. Briefly, after a given incubation the cells were incubated with 1 mL MTT (0.5 mg/mL in IB) for further 1.5 h at 37 °C. After removing the supernatant, the formazane generated by the cells was dissolved in 500 µL DMSO. 50 µL of this solution was diluted with 150 µL DMSO and the absorbance was measured at 540 nm in wells in a microtiter plate reader (Tecan, Grödig, Austria). The extracellular lactate content in the medium was determined by an enzymatic assay also in microtiter plates as described previously [27,28].

### 3.5. Experiments on Macrostomum Lignano

The culture of *Macrostomum lignano* was originally received from Dita Vizoso and Lucas Schärer (Basel) and was raised and maintained in 16/8 LD cycle in Petri dishes together with the diatom *Nitzschia* sp. at 20 ± 2 °C in our lab since 2011. Worms were incubated in F/2 medium for 1 h with 20 µM LysoGlow84 and/or 60–100 nM LysoTracker Red, washed with medium, and anesthetized with 7.18% MgCl_2_.

### 3.6. Fluorescence Microscopy

LysoGlow84 fluorescence in primary astrocyte cultures was monitored by using an Eclipse TE-2000U microscope with a DS-QilMc camera (Nikon, Düsseldorf, Germany) using appropriate filter sets (excitation: 330–380 nm, emission: 420 nm, dichromatic mirror: 400 nm). 

Co-localization of LysoGlow84 and LysoTracker Red in living cells and living individuals of *Macrostomum lignano* was monitored with a confocal laser scanning microscope TCS SP5 (Leica, Wetzlar, Germany) using appropriate excitation for the cellular fluorescence for LysoGlow84 (excitation: 340 nm, emission: 430–470 nm) and LysoTracker^®^ Red DND-99 (excitation: 561 nm, emission: 590 nm). Fluorescence spectra were measured using a Luminescence Spectrometer LS50b/Perkin Elmer.

### 3.7. Presentation of Data

Quantitative data of viability tests on astrocytes are presented as means ± SD of values that were obtained from experiments on three independently prepared cultures. The analysis of significance between groups of data was performed by ANOVA followed by the Bonferroni post hoc test with *p* > 0.05 considered as not significant. The images documenting cellular fluorescence of LysoGlow84-treated astrocytes were derived from a representative experiment that was reproduced at least once on independently prepared cultures. Fluorescence images of worms are from representative experiments that were reproduced with similar outcome at least 5 times. 

## 4. Conclusions

LysoGlow84 (Figure 1, structure 2) was chemically synthesized, its structure confirmed by mass spectrometry and NMR spectroscopy, and its fluorescent properties analyzed. The strong fluorescence of LysoGlow84 at slightly acidic pH makes it a good tool to investigate the localization of acidic cellular structures. Indeed, in cultured viable rat astrocytes and worm cells, LysoGlow84 stained cellular compartments and structures largely similar, but not identical to, staining with LysoTracker^®^ Red DND-99. This demonstrates that LysoGlow84 is a new promising dye that stains acidic compartments including lysosomes.

## References

[1] Haddock S.H., Moline M.A., Case J.F. (2010). Bioluminescence in the sea. Ann. Rev. Mar. Sci..

[2] Clark G.L., James H.R. (1939). Laboratory analysis of the selective absorption of light by seawater. J. Opt. Soc. Am..

[3] Fujita M., Nakao Y., Matsunaga S., Seiki M., Itoj Y., Yamashita J., van Soest R.W.M., Fusetani N. (2003). Ageladine A: An anti angiogenetic matrix metalloproteinase inhibitor from the marine sponge *Agelas nakamurai*. J. Am. Chem. Soc..

[4] Bickmeyer U., Grube A., Klings K.W., Köck M. (2008). Ageladine A, a pyrrole-imidazole alkaloid from marine sponges, is a pH sensitive membrane permeable dye. Biochem. Biophys. Res. Commun..

[5] Bickmeyer U. (2012). The alkaloid ageladine A, originally isolated from marine sponges, used for pH-sensitive imaging of transparent marine animals. Mar. Drugs.

[6] Bickmeyer U., Heine M., Podbielski I., Münd D., Köck M., Karuso P. (2010). Tracking of fast moving neuronal vesicles with ageladine A. Biochem. Biophys. Res. Commun..

[7] Shengule S.R., Karuso P. (2006). Concise total synthesis of the marine natural product ageladine A. Org. Lett..

[8] Meketa M.L., Weinreb S.M. (2006). Total synthesis of ageladine A, an angiogenesis inhibitor from the marine sponge *Agelas nakamurai*. Org. Lett..

[9] Ando N., Terashima S. (2009). Synthesis of novel ageladine a analogs showing more potent matrix metalloproteinase (MMP)-12 inhibitory activity than the natural product. Bioorganic Med. Chem. Lett..

[10] Appelqvist H., Waster P., Kagedal K., Ollinger K. (2013). The lysosome: From waste bag to potential therapeutic target. J. Mol. Cell Biol..

[11] Ladurner P., Schärer L., Salvenmoser W., Rieger R.M. (2005). A new model organism among the lower Bilateria and the use of digital microscopy intaxonomy of meiobenthic Platyhelminthes: *Macrostomum lignano*, n. sp. (Rhabditophora, Macrostomorpha). J. Zool. Syst. Evol. Res..

[12] Rivera-Ingraham G.A., Bickmeyer U., Abele D. (2013). The physiological response of the marine platyhelminth *Macrostomum lignano* to different environmental oxygen concentrations. J. Exp. Biol..

[13] Tietje K., Rivera-Ingraham G., Petters C., Abele D., Dringen R., Bickmeyer U. (2013). Reporter dyes demonstrate functional expression of multidrug resistance proteins in the marine flatworm *Macrostomum lignano*: The sponge-derived dye Ageladine A is not a substrate of these transporters. Mar. Drugs.

[14] Shengule S.R., Loa-Kum-Cheung W.L., Parish C.R., Blairvacq M., Meijer L., Nakao Y., Karuso P. (2011). A one-pot synthesis and biological activity of ageladine A and analogues. J. Med. Chem..

[15] Ando N., Terashima S. (2007). Synthesis and matrix metalloproteinase (MMP)-12 inhibitory activity of ageladine A and its analogs. Bioorganic Med. Chem. Lett..

[16] Ma Y., Nam S., Jove R., Yakushijin K., Horne D.A. (2010). Synthesis and anticancer activities of ageladine A and structural analogs. Bioorganic Med. Chem. Lett..

[17] Van den Eynde J.J., Delfosse F., Mayence A., Van Haverbeke Y. (1995). 2,3-Dichloro-5,6-dicyano-1,4-benzoquinone, a mild catalyst for the formation of carbon-nitrogen bonds. Tetrahedron.

[18] Vicente-Garcia E., Ramón R., Preciado S., Lavilla R. (2011). Multicomponent reaction access to complex quinolines via oxidation of the Povarov adducts. Beilstein J. Org. Chem..

[19] Karuso P., Shengule S.R. (2009). Synthesis of Ageladine a and Analogs Thereof. Patent.

[20] Freundt E., Czapiga M., Lenardo M. (2007). Photoconversion of LysoTracker^®^ Red DND-99 to a green fluorescent molecule. Cell Res..

[21] Thein T., Mariani F. (2012). Use of LysoTracker to detect programmed cell death in embryos and differentiating embryonic stem cells. J. Vis. Exp..

[22] Wiegand U.K., Duncan R.R., Greaves J., Chow R.H., Shipston M.J., Apps D.K. (2003). Red, yellow, green go!—A novel tool for microscopic segregation of secretory vesicle pools according to their age. Biochem. Soc. Trans..

[23] Guillard R.R.L., Ryther J.H. (1962). Studies on marine planktonic diatoms. I. *Cyclotella*680 *nana* Hustedt and *Detonula confervaceae* (Cleve). Gran. Can. J. Microbiol..

[24] Hamprecht B., Löffler F. (1985). Primary glial cultures as a model for studying hormone action. Methods Enzymol..

[25] Tulpule K., Hohnholt M.C., Hirrlinger J., Dringen R., Hirrlinger J., Waagepetersen H. (2014). Primary cultures of astrocytes and neurons as model systems to study the metabolism and metabolite export from brain cells. Neuromethods 90: Brain Energy Metabolism.

[26] Scheiber I.F., Schmidt M.M., Dringen R. (2010). Zinc prevents the copper-induced damage of cultured astrocytes. Neurochem. Int..

[27] Dringen R., Gebhardt R., Hamprecht B. (1993). Glycogen in astrocytes: Possible function as lactate supply for neighboring cells. Brain Res..

[28] Liddell J.R., Zwingmann C., Schmidt M.M., Thiessen A., Leibfritz D., Robinson S.R., Dringen R. (2009). Sustained hydrogen peroxide stress decreases lactate production by cultured astrocytes. J. Neurosci. Res..

